# Plant electrophysiological responses to drought and their association with live fuel moisture and flammability in *Salvia rosmarinus*

**DOI:** 10.1080/15592324.2026.2707675

**Published:** 2026-08-10

**Authors:** David Fuente, Rodolfo Zapata, Ernesto Martínez-Heras, Maria Dolores Raigón, Jose-Vicente Oliver-Villanueva

**Affiliations:** a ITACA – Institute of Information and Communication Technologies, Universitat Politècnica de València, València, Spain; b Instituto de Conservación y Mejora de la Agrobiodiversidad Valenciana (COMAV), Universitat Politècnica de València, Valencia, Spain

**Keywords:** *Salvia rosmarinus*, mediterranean shrublands, plant water status, electrophysiology, bio-indicator, real-time monitoring

## Abstract

Live fuel moisture content is a key determinant of live fuel flammability, yet its destructive and discontinuous measurement limits high-temporal-resolution monitoring. This study evaluated whether leaf electrical potential can serve as a non-invasive proxy for LFMC and flammability-related traits under natural drought conditions. From February to July 2025, leaf and trunk electrical potentials were monitored weekly in *Salvia rosmarinus* individuals from a Mediterranean shrubland, while LFMC, essential oil yield, fatty-acid fraction, and laboratory-based flammability metrics—ignition time, combustion duration, and flame height—were assessed bi-weekly. Leaf electrical potential was strongly associated with LFMC (R^2^ = 0.64, *p* < 0.001), decreasing as plants underwent seasonal drought-induced dehydration. Periods of high temperature and low rainfall reduced both LFMC and electrical potential, coinciding with shorter ignition times, which declined to approximately 20–30 s during the driest period. Based on the observed shifts in ignition time, combustion duration, and flame height, three empirical LFMC response zones were identified, with leaf electrical potential closely tracking transitions in plant hydration and flammability. These results suggest that plant electrophysiology may provide a promising non-invasive indicator of live fuel water status and seasonal flammability dynamics, with potential applications in wildfire risk monitoring when combined with conventional LFMC, meteorological, and remote-sensing approaches.

## Introduction

Mediterranean climates are characterized by the coincidence of the dry season with peak annual temperatures, rendering vegetation highly flammable and particularly susceptible to summer wildfires.^1^ While large-scale fires have historically shaped vegetation dynamics across Mediterranean-type ecosystems—including the Mediterranean Basin, California, Central Chile, the Cape Region of South Africa, and southern Australia—traditional agro-silvo-pastoral practices previously maintained landscape fragmentation, thereby mitigating fire risk.^2^ However, widespread rural depopulation since the mid-20th century has driven vegetation recovery on abandoned croplands across the Mediterranean Basin.^3^ Successional processes in these lands often lead to the dominance of shrub species such as rosemary (*Salvia rosmarinus*), especially on limestone soils, where it displaces pioneer species over time.^4^ ^,^ ^5^ Studies have linked this land abandonment to increased burned areas and accelerated fire spread, a trend particularly evident in Spain.^6^ Post-fire dynamics further reinforce this shrub dominance, as observed in eastern Spain, where *Salvia rosmarinus* becomes structurally prominent decades after abandonment and disturbance.^7^ This landscape homogenization, coupled with rising temperatures and increased human activity, has contributed to a marked rise in fire frequency and total burned area in recent decades.^8^

Current wildfire risk assessment across the Mediterranean basin largely relies on the Fire Weather Index (FWI). While effective, this and similar systems are predominantly based on meteorological variables. As ecosystems become increasingly vulnerable to extreme fire behavior, improving predictive accuracy requires moving beyond purely climatic approaches. The exclusion of critical variables—such as vegetation type, structural architecture, physiological status, and fuel moisture content, alongside topographic and micro-topographic features—significantly limits the precision of current models.^9^ These factors directly dictate fuel availability and distribution, thereby influencing ignition probability and fire spread rates. Moreover, applying uniform risk levels across large regions obscures fine-scale variability, often resulting in generalized, less effective alerts.^10^

To address these limitations, researchers have long utilized live fuel moisture content (LFMC) as a proxy for vegetation status. Considerable effort has been devoted to estimating LFMC through gravimetric sampling,^11^ remote sensing techniques,^12^ meteorological drought indices,^13^ and empirical fire spread models.^14^ While spectral data from satellite imagery has enabled landscape-scale mapping of LFMC,^15^ these estimates often suffer from coarse spatial resolution, sensitivity to atmospheric interference, and uncertainties introduced by vegetation heterogeneity. Consequently, there is a growing consensus that integrating biophysical models with remote sensing enhances both predictive power and ecological relevance.^16^ ^,^ ^17^ This has prompted interest in identifying biological proxies capable of capturing fuel dynamics more continuously and at finer scales.

Plant electrophysiological signals may represent such a proxy, as they emerge from a complex interplay of ionic fluxes, membrane polarization, and xylem hydraulic conductivity, reflecting the integrated physiological state of the plant.^18^ ^,^ ^19^ Establishing a robust relationship between these electrical signals and LFMC offers a promising avenue for the high-resolution, real-time monitoring of vegetation flammability. Despite growing interest, research into plant electrophysiology remains limited, particularly within Mediterranean ecosystems. Nonetheless, emerging studies suggest these signals are sensitive indicators of physiological status under natural, non-controlled conditions.^20^ ^,^ ^21^ Mediterranean shrubs, in particular, exhibit rapid physiological responses to climatic variability.^22^ Specifically, *Salvia rosmarinus* displays marked seasonal fluctuations in hydration and metabolic activity that closely track rainfall patterns^23^ and extreme heat events.^24^ Unlike drought-resilient conifers such as Pinus halepensis, which maintain relatively stable LFMC throughout the year,^25^ ^,^ ^26^ the pronounced physiological plasticity of rosemary makes it an ideal model for testing whether electrophysiological signals can reliably reflect short-term dynamics in water status and associated flammability traits.

The flammability of rosemary and other Mediterranean shrub species is determined by a complex interplay of chemical, physiological, and structural traits shaped by environmental conditions. A key driver of flammability in *Salvia rosmarinus* is its high concentration of volatile organic compounds (VOCs), particularly terpenoids, which are the main constituents of its essential oils.^27^ ^,^ ^28^ These compounds exhibit low ignition temperatures and high heat of combustion, significantly promoting rapid ignition and flame propagation.^29^ Consequently, the concentration of these compounds is a critical variable for wildfire risk assessment,^30^ and aromatic species within the Lamiaceae family are widely recognized as highly flammable components of Mediterranean landscapes.^31^ ^,^ ^32^

However, current wildfire risk assessment tools often fail to integrate these fine-scale physiological and chemical dynamics. Given the pronounced physiological plasticity of *Salvia rosmarinus* and the previously noted limitations in meteorological-based fire danger systems, this study aims to bridge this gap by investigating the efficacy of electrophysiological signals as non-invasive, high-resolution indicators of vegetation water status and flammability. Specifically, we intend to assess the relationship between electrophysiological signals and the live fuel moisture content in *Salvia rosmarinus* under natural drought conditions, evaluate the capacity of these signals to act as a reliable proxy for key flammability traits—such as ignition time and combustion behavior—and identify specific flammability thresholds linked to electrical signals, thereby providing a potential framework for improved, real-time early-warning systems for wildfire danger.

## Materials and methods

### Study site and sampling design

**Figure 1.**
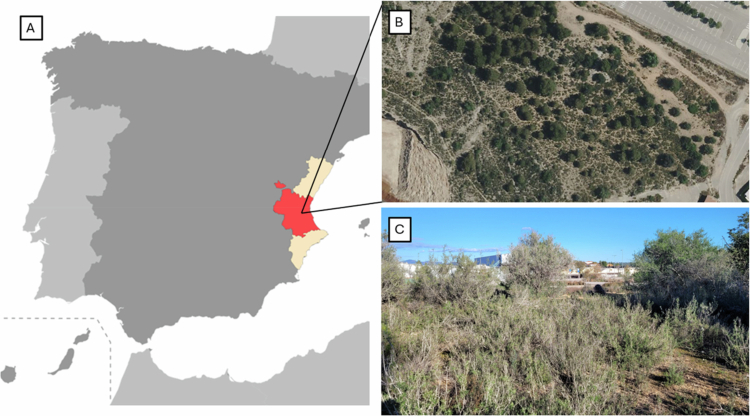
Geographic location (39°32'14"N 0°32'51"W) and habitat of the studied Salvia population. (A) Map of the Iberian Peninsula showing the location of the study area in eastern Spain, within the Valencian region. (B) Aerial view of the study site illustrating the landscape, characterized by patchy shrubland vegetation interspersed with open areas. (C) Ground-level photograph of the habitat, showing dry, open scrubland dominated by shrubs and herbaceous species.

Fieldwork was conducted on a plot of agricultural origin in Valencia (Spain) that is currently abandoned and undergoing natural regeneration with mixed forest vegetation. This situation is representative of many Mediterranean landscapes, where less productive agricultural lands have been abandoned for decades and are progressively colonized by forest species. The study plot hosts a diverse plant community, including Aleppo pine (*Pinus halepensis*), rosemary (*Salvia rosmarinus*), black hawthorn (*Rhamnus lycioides*), species of the genera Cistus and Calluna, kermes oak (*Quercus coccifera*), and gorse (*Genista sp*.). Remnant individuals of olive (*Olea europaea*) and carob (*Ceratonia siliqua*) persist from the site’s former agricultural use. The plot is located in the municipality of Riba-roja de Túria (39°32'14"N 0°32'51"W), in a representative wildland-urban interface (WUI) with high ignition and wildfire risk (Figure 1A). The study period extended from February to July 2025.

Rosemary was selected as the study species because it is a woody shrub widely distributed throughout Mediterranean-type ecosystems and commonly found in fire-prone areas. Moreover, its morphological characteristics facilitate the installation of electrophysiological sensors, and its high flammability, associated with its volatile compound content and biomass structure, makes it particularly suitable for studying plant-level drivers of fire behavior.

**Figure 2.**
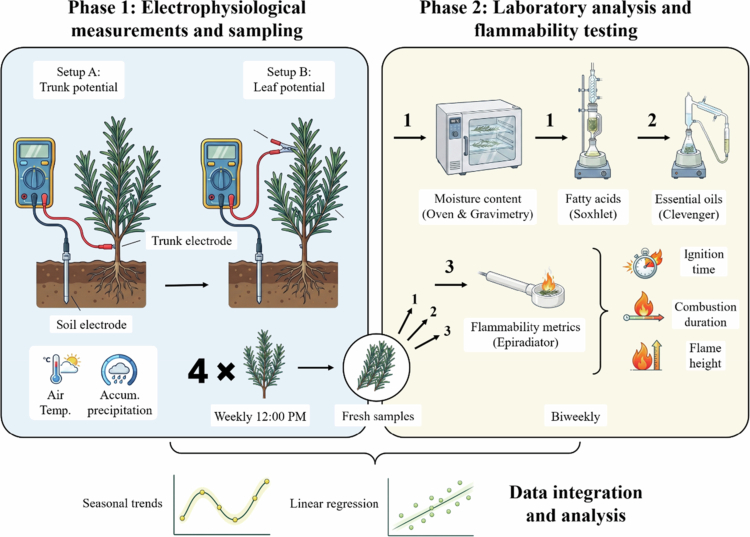
Conceptual workflow of the experimental design used to integrate field electrophysiological monitoring, laboratory biochemical analyzes, and flammability testing in Salvia rosmarinus. Fieldwork was conducted in Riba-roja de Túria, Valencia, Spain, from February to July 2025. In each sampling campaign, four rosemary individuals were monitored. Electrical potential was recorded weekly at approximately 12:00 h using a soil reference electrode, with measurements taken sequentially at two plant organs: first at the trunk and then at the leaf level. After the electrical measurements, fresh sprigs were collected from the same monitored individuals and transported to the laboratory in sealed bags to minimize dehydration. Laboratory analyzes were conducted bi-weekly using fresh leaves and included live fuel moisture content (LFMC), essential oil yield, fatty fraction yield, and flammability metrics. Three separate samples were collected from each rosemary plant: one sample was oven-dried and subsequently used for fatty acid analysis, a second sample was used for essential oil extraction with a Clevenger apparatus, and a third sample was used for the flammability experiments. This sampling scheme was applied to the four individual rosemary plants at each sampling date. LFMC was determined gravimetrically after oven drying at 90 °C for 24 h. Essential oils were extracted from fresh leaves obtained from the collected sprigs using a Clevenger apparatus for 2 h, with extractions performed in triplicate. Fatty content was determined from dried and ground leaf material using Soxhlet extraction with petroleum ether, with analyzes performed in duplicate. Flammability was assessed using approximately 1 g of fresh leaves exposed to a 500 W epiradiator, recording ignition time, combustion duration, and maximum flame height. Climatic data, including air temperature and 7-day accumulated precipitation, were obtained from a nearby validated meteorological station and integrated with electrophysiological, biochemical, moisture, and flammability data for statistical analysis.

In each sampling session, four individuals of *Salvia rosmarinus* with similar characteristics were selected. Although the number of individuals was limited due to economic resources’ restrictions, repeated measurements over time provided a longitudinal dataset capturing seasonal variability in physiological and flammability traits. The selection of plots and individuals was conducted randomly within the studied plot (Figure 1B), with plants chosen to have similar height and trunk diameter to minimize the effect of size-related variability. Plant samples were collected from the same individuals whose electrical potential was measured in the field for further biochemical and flammability characterization in the laboratory. Electrical potential was measured weekly, whereas moisture, biochemical, and flammability lab analyzes were conducted on a bi-weekly basis (Figure 2).

#### Phase 1

##### Electrophysiological analysis.

The electrophysiological analysis was conducted following a systematic workflow designed to minimize measurement artifacts and ensure data reproducibility. The acquisition process consisted of four distinct phases: (1) site preparation, (2) electrode installation, (3) signal stabilization, and (4) data acquisition.

##### Site and electrode preparation.

**Figure 3.**
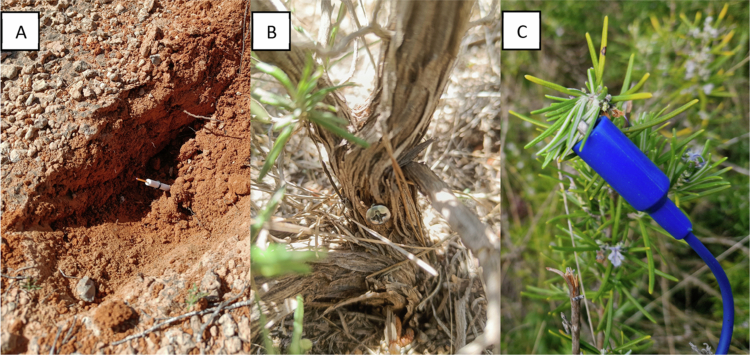
Positioning of the electrodes for measurement acquisition. (A) Ground (reference) electrode inserted into the mineral soil after removing the superficial organic layer to ensure stable electrical contact with the soil. (B) Trunk electrode placed at the basal woody stem, inserted into the conductive tissue to record electrical potential representative of the main plant axis. (C) Leaf electrode positioned on a healthy, fully developed sprig to measure foliar electrical potential. This configuration allows simultaneous recording of electrical signals at different plant compartments, enabling comparison between leaf- and trunk-level electrophysiological responses to environmental conditions.

Measurements were performed on *Salvia rosmarinus* specimens, with all data collected around 12:00 h to mitigate the influence of diurnal variability in plant electrical signals. Electrical potentials were measured relative to a common ground electrode inserted into the mineral soil approx. 25 cm depth (Figure 3A). To minimize potential differences at the electrode–electrolyte interface, a non-polarizable platinum electrode was utilized as the circuit ground.^33^ For trunk measurements, 16-mm stainless-steel screw electrodes were inserted into woody tissue^34^ to ensure robust contact (Figure 3B). For leaf measurements, surgical-grade stainless steel clamp electrodes were employed to accommodate the morphology of *Salvia rosmarinus* leaves (Figure 3C).

##### Workflow protocol.

During each weekly field campaign, the following protocol was strictly observed:

Sequence of operations: To avoid potential artifacts associated with physical handling, all electrophysiological recordings were performed in vivo on intact plants. Destructive sampling or biomass collection for secondary analyzes was carried out exclusively after the electrical measurements were completed.

Installation and stabilization: Due to the fragile nature of *Salvia rosmarinus* leaves and rapid tissue turnover, long-term electrode pre-insertion was not feasible; therefore, electrodes were positioned on the day of measurement. To ensure signal reliability and eliminate transient insertion-induced artifacts (e.g., wound potentials), the electrical signal was continuously monitored post-insertion. Data acquisition commenced only once the voltage reached a stable baseline.

Data acquisition and instrumentation: Electrical signals were recorded using a UNI-T UT71D multimeter (input impedance of ~2.5 GΩ in the 200 mV DC range, 0.05% accuracy), which is consistent with the instrumentation used in previous studies of this nature. Instrument stability was re-verified at the end of each sampling session to ensure consistency.

#### Phase 2

##### Biochemical analysis.

For each sampling date, approximately 300 g of fresh biomass per plant were collected, consisting of representative sprigs that reflected the overall physiological condition of the individual (Figure 1C). The collected fresh material was subdivided for moisture determination, biochemical analysis, and flammability tests. Samples were stored in numbered, hermetically sealed bags to prevent dehydration during transport to the laboratory.

##### Essential oil determination.

Essential oils were extracted by steam distillation using a Clevenger apparatus coupled to 1 L Pyrex boiling flasks and a heating mantle appropriate for the volume.^35^ For this procedure, the rosemary sample of fresh leaves was weighed (approximately 200 g) and placed in the flask, after which distilled water was added. Once the distillation system was assembled, heating was initiated. During boiling, water vapor entrained the volatile compounds, which subsequently condensed and separated by density difference in the graduated arm of the Clevenger apparatus. The volume of oil was monitored every 15 minutes until a constant value was reached. It was determined that 2 h of distillation was sufficient to obtain the maximum yield. After extraction, the volume of essential oil obtained was measured, and the yield (mL of essential oil per g of fresh sample) was calculated (Equation (1)):

(1) $$\mathrm{Essential}\;\mathrm{oils}\;\left(\%\right)=\frac{V}{\mathrm{FW}}\times 100$$

where V is the volume (mL) of essential oil extracted and FW is the fresh weight (g) of the sample introduced into the flask. All extractions were performed in triplicate to ensure analytical reproducibility.

##### Fatty content determination.

The extraction of the fatty content of rosemary was performed using a Soxhlet method with a FOSS ST 243 Soxtec™ semi-automatic extraction system, using petroleum ether as the solvent. For this purpose, the same dry sample used for moisture content determination was employed. After drying, the leaves were finely ground and introduced into the extraction unit. The complete Soxtec™ extraction cycle includes the phases of boiling, rinsing, and solvent recovery. Once the process was completed, the extracted fraction was weighed to determine the fatty content of the sample (Equation (2)):

(2) $$\mathrm{Fatty}\;\mathrm{content}\left(\%\right)=\frac{\left(W_{1}-W_{2}\right)}{W_{0}}\times 100$$

where $W_{1}$ is the weight (g) of the flask containing the ethereal extract, $W_{2}$ is the weight (g) of the empty flask, and W is the weight (g) of the initial sample.

Each sample was analyzed in duplicate.

##### Moisture content determination.

Moisture content in lignocellulosic materials was quantified using a drying oven and a precision balance (±0.001 g). The procedure consisted of weighing a fresh sample and placing it in the oven at 90 °C for 24 h.^11^ This temperature was selected to ensure complete water removal while minimizing thermal degradation of volatile compounds. After drying, the sample was weighed again. The weight loss was attributed to the water evaporated during the drying process and was used to calculate moisture content according to the following equation:

(3) $$H=\frac{\left(\mathrm{FW}-\mathrm{DW}\right)}{\mathrm{DW}}\times 100$$

where H is the moisture content (%), FW is the fresh weight (g) of the sample, and DW is the dry weight (g) of the sample. Using this formulation, moisture content expressed on a dry-weight (anhydrous) basis may exceed 100%, depending on the species and on the proportion of leaves or needles in the sample.

##### Flammability tests.

Flammability is the propensity of a material to ignite and sustain combustion, as well as the intensity of the combustion process. In this study, flammability was evaluated using three key variables: time to ignition, duration of combustion, and maximum flame height.^36^ Time to ignition is defined as the elapsed time until combustion with a visible flame occurs. The duration of combustion corresponds to the time during which combustion is sustained until extinction, while flame height is defined as the maximum vertical height reached by the flame. To determine these parameters, a Quartzalliance 500 W epiradiator (model 534 RC 2) was used. Epiradiators constitute a cost-effective approach for flammability testing and yield reliable results comparable to those obtained with more expensive fire-testing systems.^37^ This device is equipped with a radiant disk 10 cm in diameter, delivering a radiant power of 7 W·cm^−2^ and reaching an operating temperature of approximately 600 °C. A butane torch was employed as the ignition source, providing a stable pilot flame. All experiments were conducted inside a fume hood to ensure safe operating conditions and to minimize interference from air currents.

The experimental procedure followed protocols previously described in the literature.^38^ Once the epiradiator reached its operating temperature, the pilot flame was positioned 4 cm above the center of the radiant disk. This configuration allows the generation of controlled thermal radiation conditions and provides good repeatability, making it suitable for comparative testing of multiple samples.

Once the epiradiator reached thermal stability, the pre-weighed plant sample was placed on the radiant disk. Each flammability test was conducted using approximately 1.0 g of fresh sprigs.^38^ Immediately thereafter, the pilot flame was positioned at the prescribed distance. From this moment, the time to ignition was recorded until the appearance of the first visible flame. The maximum flame height was then measured using a tape measure as a reference scale, and the total duration of combustion was also recorded. After each test (one per sampled plant), the radiant disk was allowed to cool before removing ash residues, ensuring consistent experimental conditions between successive measurements.

Each measurement was performed once per plant and sampling date, resulting in four replicates per campaign.

##### Climatic data collection.

Climate plays a key role in regulating the dynamics of physiological and biochemical parameters in plant species, directly influencing both plant water status and fuel flammability. In this study, three main climatic variables were considered: air temperature, accumulated precipitation, and relative humidity, due to their direct effects on the physiological activity of rosemary, as well as on its moisture content and the concentration of volatile and flammable compounds.

Climatic data were collected throughout the entire sampling period using a meteorological station audited and validated by the Valencian Association of Meteorology (AVAMET). The station is located within the municipality of Riba-roja de Túria, close to the study plot. Analysis of the temporal variability of these climatic factors enabled the assessment of relationships between environmental conditions and the electrical, physiological, and flammability parameters measured during the study.

##### Statistical analysis.

All analyzes were conducted using R (v4.5.1). To explore temporal patterns in physiological and fire-related traits, we first visualized their seasonal dynamics from late winter through summer. Smoothed trends were fitted using LOESS (locally estimated scatterplot smoothing) regression to highlight nonlinear trajectories while preserving local variability. These visualizations helped identify key phenological transitions and moisture declines associated with fire risk.

We also investigated the relationship between foliar electrical potential and all other physiological, biochemical, and flammability-related variables. Pairwise Pearson correlation coefficients were computed using complete-case data. Results were presented in a panel of scatterplots with linear regression lines and 95% confidence bands, summarizing the strength and direction of association between foliar electrical potential and each trait. The panels were arranged according to the strength and direction of the correlation with leaf electrical potential to facilitate interpretation.

Following the exploratory correlation analysis, we performed linear regression modeling to quantify the relationship between leaf electrical potential and LFMC. A simple linear model was fitted using ordinary least squares, with LFMC as the dependent variable and leaf voltage as the predictor. Model assumptions were tested through standard diagnostic procedures. Residual normality was assessed using Q–Q plots and the Shapiro–Wilk test. Homoscedasticity was evaluated visually through residuals versus fitted values plots and formally using the Breusch–Pagan test.

## Results

### Seasonal dynamics of electrical potential

**Figure 4.**
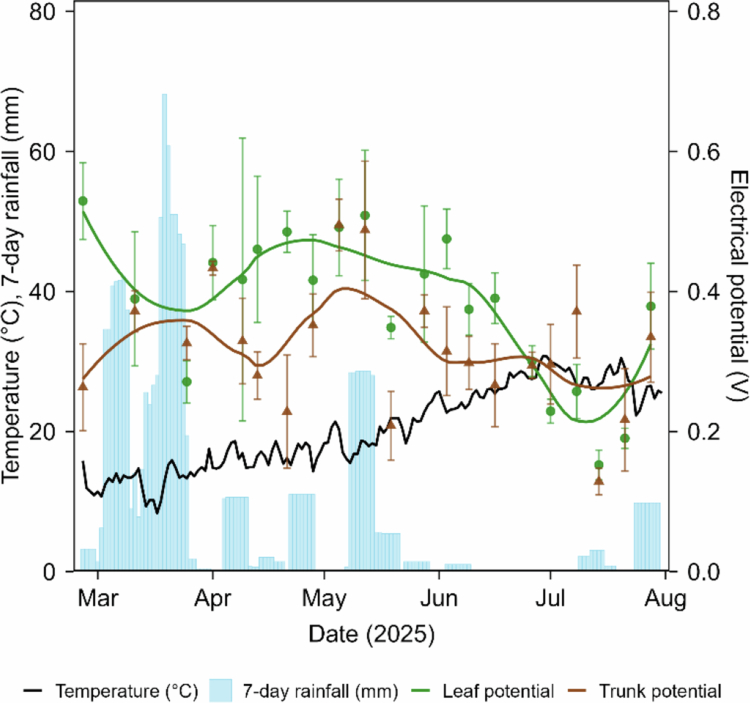
Seasonal dynamics of electrical potential in Salvia rosmarinus. Temporal evolution of electrical potential measured in rosemary leaves (green line, V) and trunks (brown line, V) from late February till the end of July 2025, together with daily mean air temperature (black line, °C) and cumulative precipitation over the preceding seven days (blue bars, mm). Points represent the mean values, with error bars indicating standard deviation for leaf (green circles) and trunk (brown triangles) measurements. Solid lines represent LOESS-smoothed trends.

To assess seasonal variation in plant bioelectrical activity, electrical potential was monitored in *Salvia rosmarinus* under field conditions throughout the study period (Figure 4). Both leaf and trunk potentials peaked in late winter and spring, coinciding with moderate temperatures, flowering, and increased precipitation. During this period, leaf potential remained mostly around 0.45–0.55 V, while trunk potential fluctuated between 0.30 and 0.40 V. Despite substantial dispersion among individual measurements, leaf potential was generally higher than trunk potential, with both signals converging only under summer conditions. From late May onward, potential values declined progressively as mean temperature increased and rainfall decreased, reaching their lowest values in July, mostly within the 0.20–0.30 V range. Notably, July rainfall events triggered a sudden increase in both leaf and trunk electrical potential, which doubled from 0.15 V to above 0.35 V.

### Flammability metrics and physiological traits

**Figure 5.**
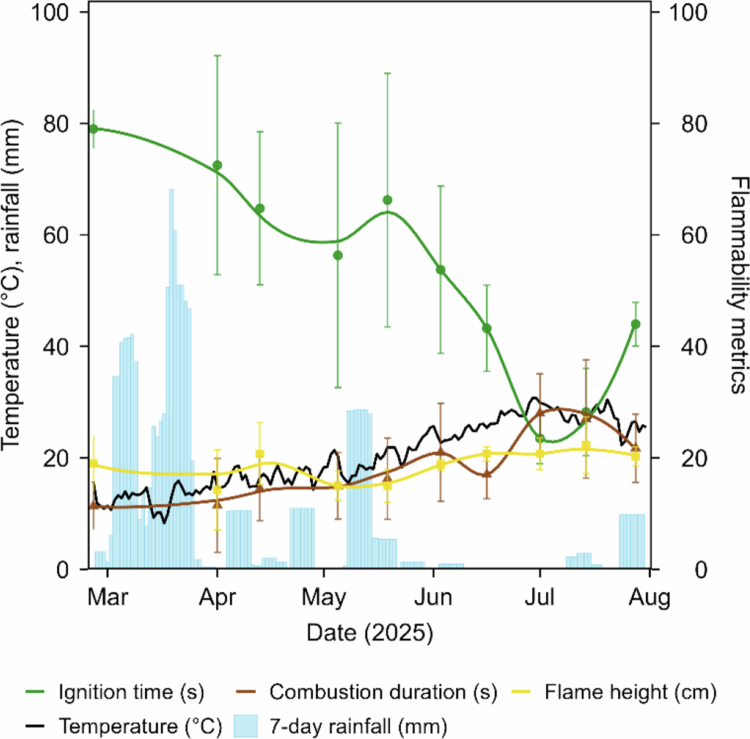
Seasonal dynamics of flammability parameters in Salvia rosmarinus. Temporal evolution of ignition time (green line, s), flame height (yellow line, cm), and combustion duration (brown line, s) obtained from epiradiator tests, together with daily mean air temperature (black line, °C) and cumulative precipitation over the preceding seven days (blue bars, mm), from late February to August 2025. Points represent mean values, with error bars indicating standard deviation (SD) for ignition time (green circles), flame height (yellow squares), and combustion duration (brown triangles). Solid lines represent LOESS-smoothed trends.

**Figure 6.**
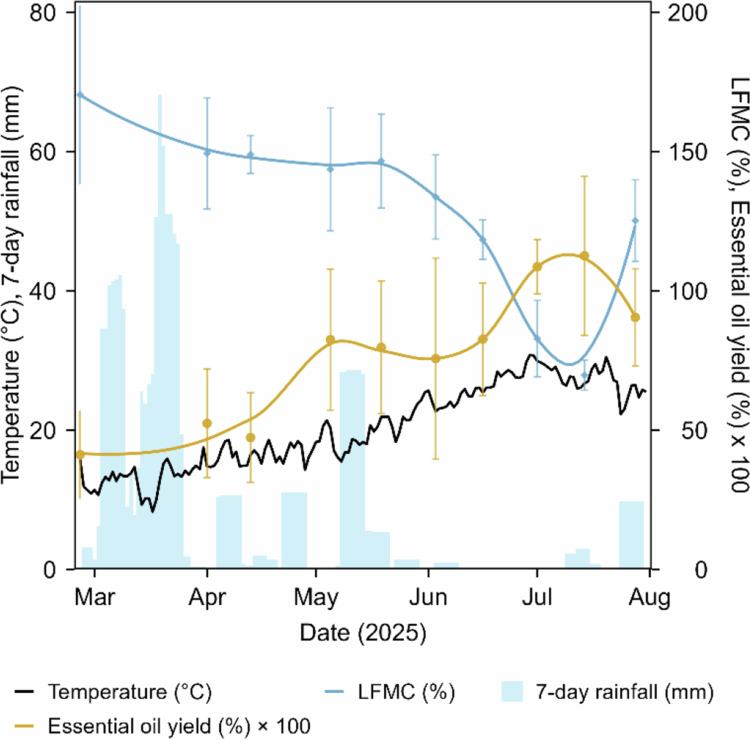
Seasonal dynamics of essential oil yield, fatty-acid fraction yield, and live fuel moisture content (LFMC) in Salvia rosmarinus. Temporal evolution of essential oil yield (gold line, %, values multiplied by 100 for visualization), and LFMC (dark blue line, %), measured from rosemary plant samples, together with daily mean air temperature (black line, °C) and cumulative precipitation over the preceding seven days (blue bars, mm), from late February to August 2025. Points represent mean values, with error bars indicating standard deviation. Solid lines represent LOESS-smoothed trends.

Flammability metrics derived from epiradiator tests revealed clear seasonal patterns (Figure 5). Ignition time ranged between 60 and 75 s during spring before showing a sharp decline from late May onward. The lowest ignition times were recorded during the summer drought period, with values falling to approximately 20–30 s. Subsequently, ignition time increased from approximately 25 s to above 40 s following July rainfall events; this recovery coincided with the observed rises in leaf electrical potential (Figure 4) and LFMC (Figure 6). This temporary increase interrupted the overall summer decline, although ignition time remained below the highest spring values.

In contrast, flame height and combustion duration exhibited more moderate seasonal trends, typically peaking during the warmest months. Combustion duration increased from approximately 15 s to 25 s in spring to around 20−25 s in summer, with the highest values recorded just before the rainy events in July. The highest combustion durations were observed between June and July, when temperatures were highest and rainfall was limited. Flame height remained comparatively stable throughout the study period, fluctuating mostly between 18 and 22 cm. Overall, the three flammability variables showed contrasting seasonal responses: ignition time decreased to approximately one-third of its spring values before the summer rainfall events, combustion duration nearly doubled, and flame height remained almost constant.

LFMC showed an overall declining seasonal trend with distinct phases, starting at approximately 170% in late winter and remaining around 145–155% in May (Figure 6). This was followed by a sharp decline to below 70% by mid-July, before a rapid recovery to over 120% following rainfall events. The steepest LFMC decrease occurred between June and mid-July, coinciding with the period of highest temperatures and lowest rainfall. Regarding secondary metabolites, essential oil yield increased steadily from 0.5% (w/w) in March to a maximum of around 1.2% (w/w) in mid-July. The highest essential oil values were recorded during the same period in which LFMC reached its minimum values. Conversely, fatty acid yield remained stable between 0.1% and 0.2% (w/w) with no clear temporal trend (data not shown).

### Flammability regimes along moisture and electrical gradients

**Figure 7.**
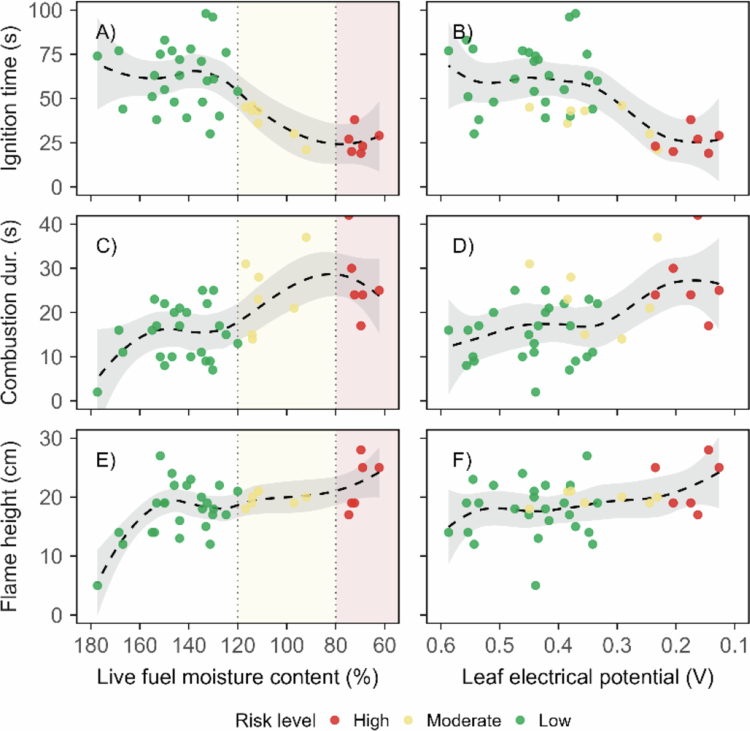
Fire-related flammability metrics of Salvia rosmarinus as a function of plant water status and leaf electrical potential. Panels A, C, and E show ignition time (s), combustion duration (s), and flame height (cm) plotted against live fuel moisture content (LFMC, %), while panels B, D, and F show the same variables as a function of leaf electrical potential (V). In panels A, C, and E, the x-axis is inverted so that drier conditions appear to the right. Points represent individual epiradiator test measurements and are colored by LFMC-based fire-risk classes. Dashed lines show LOESS smoothed trends, and shaded bands indicate 95% confidence intervals. In panels A, C, and E, vertical dotted lines mark LFMC thresholds, and background shading denotes empirical fire activity categories: low (LFMC > 120%), moderate (80% < LFMC ≤ 120%), and high (LFMC ≤ 80%).

Figure 7 shows how key flammability metrics varied across gradients of LFMC (Figure 7A, C, E) and leaf electrical potential (Figure 7B, D, F). To summarize the observed response patterns, LFMC values were grouped into three post hoc empirical flammability zones: high LFMC (>120%), intermediate LFMC (80–120%), and low LFMC (≤80%). These classes were not intended to represent universal fire-risk thresholds, but to describe the main transitions observed in *Salvia rosmarinus* under the conditions of this experiment.

Across the high-LFMC range, ignition time remained relatively stable at approximately 65–70 s. In contrast, combustion duration and flame height showed a biphasic pattern: both metrics initially increased at the highest LFMC values (150–180%), followed by a plateau at approximately 15 s and 18 cm, respectively, within the 120–150% LFMC interval. As LFMC declined into the intermediate range, ignition time decreased markedly, while combustion duration and flame height increased. Under low-LFMC conditions (≤80%), ignition time stabilized at low values of approximately 20–30 s, representing a near threefold reduction compared with wetter conditions. Combustion duration reached up to approximately 30 s, and flame height increased to around 25 cm.

When plotted against leaf electrical potential, the flammability metrics showed broadly similar trends to those observed along the LFMC gradient, although with greater dispersion and more overlap among LFMC classes (Figure 7B, D, F). Ignition time showed the clearest response: values remained generally high at leaf potentials above approximately 0.35–0.40 V, but declined sharply as electrical potential decreased, reaching minimum values under low-voltage conditions (Figure 7B). Combustion duration followed the opposite pattern, with relatively low and variable values at higher electrical potentials and a marked increase as potentials declined below approximately 0.30 V (Figure 7D). Flame height showed a weaker response than the other two metrics, with substantial scatter across the voltage gradient, although the highest values tended to occur at lower electrical potentials (Figure 7F). Unlike the LFMC gradient, neither combustion duration nor flame height exhibited a biphasic pattern under high electrical potential conditions; instead, both remained relatively constant. Overall, lower leaf electrical potential was associated with shorter ignition times and greater combustion activity, but the separation among flammability classes was less distinct than along the LFMC gradient.

### Relationship between electrical potential and functional traits

**Figure 8.**
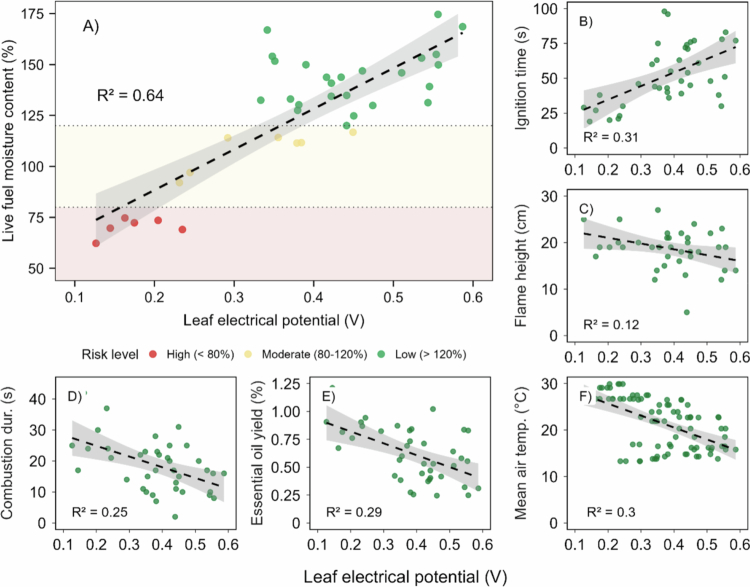
Relationship between leaf electrical potential and selected climatic, biophysical, and biochemical variables in Salvia rosmarinus. Panel (A) shows the relationship between leaf electrical potential and live fuel moisture content, including ad hoc fire risk thresholds (colored bands: red, LFMC < 80%; yellow, 80% ≤ LFMC ≤ 120%; white, LFMC > 120%). Panels (B–F) present linear regressions between leaf electrical potential and ignition time, flame height, combustion duration, essential oil yield, and mean air temperature, respectively. Shaded areas represent 95% confidence intervals. The coefficient of determination (R^2^) is shown within each panel. Panels are ordered from the strongest positive to the strongest negative association. Notably, LFMC exhibits the strongest relationship with leaf electrical potential.

Figure 8 illustrates the relationship between leaf electrical potential and various functional traits. The strongest association was observed with LFMC (R^2^ = 0.64, *p* = 6.6·10^−10^; Figure 8A), which showed a significant positive linear relationship. Residual diagnostics (Breusch–Pagan *p* = 0.875) indicated no heteroscedasticity; while residuals deviated slightly from normality (Shapiro–Wilk *p* = 0.015), their symmetric distribution suggests that the linear model provided an acceptable fit.

Leaf electrical potential decreased as water content declined across the full range (approximately 60–175% LFMC). Most observations under low flammability conditions (LFMC > 120%) exhibited potentials above 0.4 V, whereas lower values were associated with moderate-to-high flammability conditions. High-flammability observations (LFMC < 80%) were concentrated at leaf electrical potentials below approximately 0.25 V, while moderate values (80–120% LFMC) occupied an intermediate range. This transition aligns with the range where flammability metrics—such as ignition time, combustion duration, and flame height—begin to rapidly shift (Figure 8B–F). Other variables showed moderate associations with leaf voltage, including positive relationships with ignition time (R^2^ = 0.31, *p* = 2.7·10^−4^) and negative relationships with combustion duration (R^2^ = 0.25, *p* = 1.4·10^−3^), essential oil yield (R^2^ = 0.29, *p* = 3.0·10^−4^), and mean air temperature (R^2^ = *p* = 3.9·10^−8^). Flame height showed limited sensitivity, although the relationship remained statistically significant at the 5% significance level (R^2^ = 0.12, *p* = 0.036).

## Discussion

The seasonal dynamics of electrical potential observed in *Salvia rosmarinus* were closely associated with changes in plant water status and flammability. Leaf and trunk electrical potentials declined progressively from spring to summer, coinciding with increasing temperature, reduced rainfall, and a marked decrease in LFMC. This pattern suggests that the bioelectrical signal captured the progressive dehydration of live fine fuels during the seasonal drought. Electrical signals in plants are generated through changes in membrane potential and ion fluxes, and are involved in the systemic coordination of physiological responses to environmental variation.^18^ In woody plants, electrical potential dynamics have also been linked to water availability, light–dark cycles, and stress-related changes in plant status.^34^ ^,^ ^39^ In this context, the seasonal decline observed in *Salvia rosmarinus* is best interpreted as an integrated bioelectrical response to changing hydration status rather than as an isolated response to short-term meteorological variation. In this context, the sharp rise in electrical potential following the July rainfall events further indicates that the bioelectrical signal is sensitive to short-term changes in plant hydration, at least under drought-stress conditions. The seasonal dynamics of electrical potential should not be interpreted solely as a passive response to climate, but rather as part of an integrated physiological response to changing environmental conditions. As argued by Galviz et al.^40^ plant stress is not an isolated event, but a dynamic process in which prior exposure may influence subsequent physiological responses through priming and acclimation mechanisms. In this context, the rapid recovery of electrical potential following July rainfall suggests that *Salvia rosmarinus* can rapidly adjust its physiological status after rehydration, a response that was effectively captured by the bioelectrical monitoring system.

A central objective of this study was to evaluate whether leaf electrical potential could reflect seasonal changes in live fuel hydration. The strongest relationship observed was between leaf electrical potential and LFMC (R^2^ = 0.64; *p* < 0.001), indicating that bioelectrical measurements may provide a useful complementary indicator of live fuel water status. LFMC provides a direct measure of live fuel hydration, with well-documented effects on fire behavior.^41^ However, conventional LFMC measurement is destructive, discontinuous, and limited in temporal resolution. In contrast, electrical potential can be monitored non-invasively and may provide continuous information on plant physiological status. Similar approaches have recently been proposed in Mediterranean fire-prone systems, where electrical responses have been explored as complementary indicators of wildfire risk alongside LFMC.^19^ In addition, recent work in other species has shown that electrical impedance spectroscopy and continuous bioelectrical monitoring can detect drought-related physiological changes before visible foliar damage occurs.^42^ ^,^ ^43^ In our case, the positive relationship between leaf electrical potential and LFMC suggests that declining voltage reflected a progressive loss of tissue hydration, consistent with the seasonal transition toward more flammable live fuels.

Furthermore, the relationship between electrical potential and the flammability metrics supports this interpretation. Higher leaf electrical potentials were generally associated with higher LFMC and longer ignition times, whereas lower potentials were associated with shorter ignition times, longer combustion duration, and higher essential oil yield. This indicates that electrical potential tracked the seasonal transition from hydrated, less ignitable live fuel toward drier and more combustible material. However, the strength of these relationships differed among flammability variables. Ignition time showed the clearest response to changes in LFMC and electrical potential, whereas flame height was almost not correlated with leaf electrical potential. This indicates that ignition time may be the most responsive flammability metric for detecting seasonal changes in live fuel hydration in *Salvia rosmarinus*, while combustion duration and flame height may integrate additional effects of fuel structure, volatile compounds, and combustion dynamics.

In this regard, the seasonal increase in essential oil yield adds an important chemical dimension to the flammability patterns observed in this study. Essential oil yield increased during the hottest and driest period, reaching its maximum when LFMC was lowest and electrical potential had declined. Seasonal variation in rosemary essential oil yield and composition has been reported previously under Mediterranean conditions.^44^ Comparable studies also show that extraction yields vary strongly with leaf water status and drying procedure. For instance, Melese et al. ^45^ reported yields of up to 0.96% (v/w) in fresh rosemary leaves, a value close to the maximum yield obtained in our study, whereas Arpiwi and Suarni^46^ found lower yields of 0.36% in fresh leaves. This is relevant because volatile secondary compounds, particularly terpenes, can enhance flammability in rosemary and other Mediterranean species.^27^ Nevertheless, the role of essential oils should be interpreted as modulatory rather than as the sole driver of flammability. Indeed, experimental work on Mediterranean species suggests that terpenoids can contribute to flammability, but their effect depends on fuel moisture, heat flux, species identity, and the specific chemical fractions involved.^47^ In the present study, the summer increase in essential oil yield likely interacted with declining LFMC to enhance combustion duration and, to a lesser extent, flame height, while the sharp reduction in ignition time was more directly associated with the loss of live fuel moisture. In contrast, the fatty-acid fraction remained relatively stable throughout the study period and showed no clear temporal pattern. This suggests that fatty-acid yield was less responsive to seasonal drought than essential oil yield and probably played a marginal role in the observed changes in flammability.

The LFMC ranges used in this study should not be interpreted as universal flammability thresholds, but as empirical response zones derived from the observed behavior of *Salvia rosmarinus* in the flammability tests. In our study, the >120%, 80–120%, and ≤80% LFMC ranges were defined post hoc from the observed shifts in ignition time, combustion duration, and flame height, and should therefore be interpreted as species- and experiment-specific flammability response zones rather than universal fire-danger thresholds, which depend on different factors. Nevertheless, our assumed ranges are supported by literature. In Mediterranean shrubs, including *Rosmarinus officinalis*, ignition delay times have been reported to strongly correlate with LFMC.^48^ Similarly, studies in chaparral ecosystems have identified critical live fuel moisture values close to 79% in relation to large-fire occurrence,^49^ while previous fire-behavior studies have proposed LFMC values around 75% as relevant limits for fire spread in shrublands.^50^ The upper LFMC boundary of 120% is also consistent with studies reporting lower fire hazard or reduced fire intensity above this range in shrubland fuels.^51^ ^,^ ^52^ Controlled ignition experiments further show that fuel moisture strongly affects ignition processes, although the response may be modified by fuel structure, tissue properties, and chemical composition.^41^ Thus, the LFMC classes used in this work are not arbitrary. They summarize the main transitions observed in ignition and combustion behavior in the present study, while remaining consistent with previously reported LFMC ranges associated with increased ignitability and fire activity.

Overall, our results support a multi-trait interpretation of seasonal flammability in *Salvia rosmarinus*. The transition toward higher flammability was not associated with a single variable, but with the simultaneous decline in LFMC and electrical potential, the reduction in ignition time, the increase in combustion duration, and the seasonal accumulation of essential oils. This integrated response highlights the value of combining physiological, electrical, biochemical, and combustion-based measurements when assessing live fuel flammability. These results fit within the broader framework of pyro-ecophysiology, which emphasizes that live fuel flammability is controlled not only by moisture content, but also by plant physiological traits, hydraulic function, tissue structure, and chemistry.^53^ Recent studies have shown that LFMC dynamics are strongly influenced by plant functional and hydraulic traits, including water potential, capacitance, turgor-related traits, and tissue water storage capacity.^54^ ^,^ ^55^ Although these variables were not measured directly in the present study, the close relationship between electrical potential and LFMC suggests that leaf voltage may integrate several components of plant water status, including hydration, ionic balance, and tissue-level physiological activity. Within this framework, leaf electrical potential appears to be a promising non-invasive proxy for tracking seasonal changes in live fuel status, particularly when combined with direct LFMC measurements and flammability tests.

## Conclusions

This study characterizes the seasonal coupling between electrophysiological signals, live fuel moisture content, and flammability-related traits in *Salvia rosmarinus* during spring and summer 2025. Leaf electrical potential declined progressively throughout the dry season and closely tracked reductions in LFMC, showing significant associations with ignition time, combustion duration, and flame height. The increased flammability observed during the driest period may therefore be attributed to the combined effects of fuel dehydration and volatile compound accumulation, as reflected by lower LFMC and higher essential oil content. Conversely, rainfall events in July promoted rapid physiological recovery, evidenced by concurrent increases in electrical potential, LFMC, and ignition time.

The strong relationship between electrical potential and LFMC supports the use of electrophysiological signals as integrative, non-invasive indicators of plant functional status and live fuel condition. In combination with remote sensing, climatic data, and direct water-status measurements, plant-level electrical signals could improve both the temporal resolution and mechanistic interpretation of wildfire risk assessment. Further studies across multiple years, sites, species, and drought intensities will be needed to evaluate the robustness and transferability of this approach.

By integrating bioelectrical dynamics, LFMC, flammability traits, and essential oil content, this study contributes to a pyro-ecophysiological understanding of aromatic Mediterranean shrubs and suggests that electrical potential may capture broader functional transitions associated with drought-induced increases in live fuel flammability.
